# Incorporating biological knowledge in the search for gene × gene interaction in genome-wide association studies

**DOI:** 10.1186/1753-6561-3-s7-s81

**Published:** 2009-12-15

**Authors:** Alisa K Manning, Julius Suh Ngwa, Audrey E Hendricks, Ching-Ti Liu, Andrew D Johnson, Josée Dupuis, L Adrienne Cupples

**Affiliations:** 1 School of Public Health, Boston University, 715 Albany Street, Boston, Massachusetts 02118, USA; 2 National Heart Lung and Blood Institute's The Framingham Heart Study, 73 Mt. Wayte Avenue, Suite 2, Framingham, Massachusetts 01702, USA

## Abstract

We sought to find significant gene × gene interaction in a genome-wide association analysis of rheumatoid arthritis (RA) by performing pair-wise tests of interaction among collections of single-nucleotide polymorphisms (SNPs) obtained by one of two methods. The first method involved screening the results of the genome-wide association analysis for main effects *p*-values < 1 × 10^-4^. The second method used biological databases such as the Gene Ontology and Kyoto Encyclopedia of Genes and Genomes to define gene collections that each contained one of four genes with known associations with RA: *PTPN22*, *STAT4*, *TRAF1*, and *C5*. We used a permutation approach to determine whether any of these SNP sets had empirical enrichment of significant interaction effects. We found that the SNP set obtained by the first method was significantly enriched with significant interaction effects (empirical *p *= 0.003). Additionally, we found that the "protein complex assembly" collection of genes from the Gene Ontology collection containing the *TRAF1 *gene was significantly enriched with interaction effects with *p*-values < 1 × 10^-8 ^(empirical *p *= 0.012).

## Introduction

Genome-wide association studies (GWAS) are routinely used in the search for genes associated with complex traits. Often, a test of association with a phenotype of interest is performed for each single-nucleotide polymorphism (SNP) independently and pair-wise tests of interaction are used to find gene × gene interaction. In GWAS, performing all pair-wise interaction tests can be an intractable task, especially if logistic regression with covariate adjustments is used. It is imperative to develop strategies to search out gene × gene interaction in the context of GWAS. In this paper, we apply two screening approaches to reduce the number of tests performed.

The strongest known genetic association with rheumatoid arthritis (RA) is the *HLA-DRB1 *locus, which is part of the major histocompatibility complex on chromosome 6. After performing a GWAS analysis that adjusted for SNPs capturing the strong association signal from the *HLA-DRB1 *locus, we used a permutation approach to evaluate sets of candidate SNPs to determine if these sets yielded an excess of significant main effects and SNP × SNP interaction effects over what would be expected by chance.

We defined candidate sets of SNPs using two methods. The first method, suggested by Kooperberg and Leblanc [1], tests for pair-wise interaction between SNPs with significant main effects, defined here as having main effect *p*-values less than 1 × 10^-4^. In the second method, we used biological processes to define gene collections and searched for significant main effects and pair-wise interaction effects among SNPs near these genes. We sought to evaluate this gene-based approach in collections containing at least one of four genes having known associations with RA: *PTPN22 *[2,3], *STAT4 *[4], *TRAF1*, and *C5 *[5]. After a set of SNPs from either of these two methods was found to have empirical enrichment of significant interaction effects in models unadjusted for the *HLA-DRB1 *locus, we then evaluated pairwise interactions adjusting for SNPs that capture the signal from the *HLA-DRB1 *locus.

## Methods

### Study sample and genotyping

The study sample for this research consists of 2,062 individuals in the North American Rheumatoid Arthritis Consortium (NARAC), 868 who had RA and 1,194 who did not. Genotyping of the sample was performed on Infinium HumanHap550 arrays, version 1.0 (Illumina) and is described elsewhere [5]. In total, genotypes for 545,080 SNPs were made available to our study. The analysis of this data was performed as part of the Genetic Analysis Workshop 16.

The study sample and genotype data were cleaned using utilities in the PLINK software package, version 1.02 [6]. Individuals were removed from the sample if their reported sex did not match their estimated sex given their genotypes on the X chromosome. Additional individual-level cleaning consisted of checking the number of missing genotypes per person, a test of missingness by case-control status, and a haplotype test for non-random missing genotypes with respect to case-control status. SNPs were removed from the analysis if the call rate was less than 95%, the minor allele frequency was less than 1%, the Hardy-Weinberg equilibrium *p*-value was less than 10^-4^, or a test for differential missingness between cases and controls resulted in a *p*-value < 10^-4^. After filtering, the sample consisted of 2,055 individuals and 495,766 SNPs.

### Statistical analysis

Statistical analyses were performed using PLINK, version 1.02 [6] and the statistical package R, version 2.6.1. The association between each SNP and RA was assessed with logistic regression adjusting for sex and specific genetic loci as described below. In the case of the gene-centric analysis, we defined sets of SNPs as described below and used PLINK to perform tests of main effects and interaction with these SNPs. The pair-wise tests of interactions for the permutations described below were performed using the--*fast-epistasis *command in PLINK. Sets of SNPs that were enriched with significant pair-wise interactions were further evaluated using logistic regression models in R, which included SNPs explaining the association signals in the HLA-DRB1 locus.

### Adjusting for *HLA-DRB1*

It is well known that specific alleles at the *HLA-DRB1 *locus in 6p21 are strongly associated with RA. The literature suggests two classes of alleles: the highest risk alleles are DRB1*0401, *0404*, *0405*, *0408*, and *0409*, and other risk alleles are DRB1*0101, *0102*, *0104*, *0105*, *1001*, *1402*, and *1406 *[7]. In our genome-wide search for significant interacting and main effects we performed analyses adjusting for the number of high risk and other risk alleles for each individual.

After adjusting for the *HLA-DRB1 *locus, highly significant SNPs remained in the 6p21 region surrounding the *HLA-DRB1 *locus. We added SNPs to our model, one at a time, until the most significant SNP on chromosome 6 was no longer in the 6p21 region.

### Enrichment of interaction effects among SNPs with significant main effects

Kooperberg and Leblanc [1] suggest that there is more power to detect gene × gene interaction effects if tests for interaction are limited to those SNPs with significant main effects, thus limiting the number of tests performed. We defined statistical significance of main effects using a *p*-value cut-off of 1 × 10^-4^, a less conservative choice than the Bonferroni significance cut-off of 0.05/545,080 = 9.17 × 10^-7^. We then performed pair-wise interaction tests among all SNPs that passed quality control filters and had statistically significant main effects. Significance of interaction *p*-values was determined with a Bonferroni correction on the number of tests performed.

In order to determine whether this set of SNPs was enriched with more significant interaction effects than would be expected by chance, we employed a permutation approach to construct a null distribution for the proportion of interaction *p*-values less than the alpha level determined by the Bonferroni correction. We permuted the phenotype and covariates of our sample 1,000 times. For each permutation, we performed a genome-wide scan of single-SNP association, chose those SNPs with *p*-values < 10^-4^, and performed pair-wise interaction tests with this set of SNPs. The null distribution from which we determine significant enrichment of interaction *p*-values was built from the number of tests with *p*-values less than the Bonferroni correction from each permutation.

### Enrichment of main and interaction effects among SNPs in gene collections

Four genes that are known to be associated with RA are *PTPN22*, *STAT4*, *TRAF1*, and *C5*. In the case of *TRAF1 *and *C5*, a SNP was found to be associated with RA and is in linkage disequilibrium with both the *TRAF1 *and *C5 *genes; the causal variant(s) among these genes have yet to be determined. We identified pathways and gene collections containing these four genes from two sources: the Kyoto Encyclopedia of Genes and Genomes (KEGG) [8-10] and the Gene Ontology (GO) project [11]. Two genes were found in KEGG pathways. The *TRAF1 *gene is in the "small cell lung cancer" pathway and *STAT4 *is in the "JAK-STAT signaling" pathway. We used GO data sets to extract sets to which our genes are annotated in the Biological Process ontology. To avoid overly narrow or broad sets, we focused on GO sets that contained at least 30, but no more than 220, genes. We considered the following GO sets: "protein amino acid dephosphorylation" for *PTPN22*; "JAK-STAT cascade" for *STAT4*; "protein complex assembly" for *TRAF1*; "activation of MAPK activity", "chemotaxis", and "inflammatory response" for the *C5 *gene.

In the analysis of each gene collection, we used a permutation approach to determine whether the gene collection was significantly enriched with *p*-values < 10^-6^, 10^-7^, and 10^-8^. The phenotypes of our sample were permuted and pair-wise tests of interaction were performed with the SNPs in the gene collection. The observed number of SNPs with main effect *p*-values < 10^-6^, 10^-7^, and 10^-8 ^was noted for each permutation, as was the number of pair-wise interaction tests with *p*-values below the same thresholds. A null distribution of both main effect and interaction *p*-values below each threshold was built from 1,000 permutations. Significant enrichment of main effect and interaction *p*-values was determined by comparing the proportion of *p*-values less than each threshold in the set of SNPs in the gene collection with the empirical null distribution at that threshold.

## Results

### Adjusting for *HLA-DRB1*

Our analysis of main effect associations of SNPs was adjusted for the *HLA-DRB1 *locus on 6p24, using two covariates indicating the number of risk alleles per person as well as five SNPs in the immediate vicinity of the gene. These SNPs, with their gene and *p*-value from the adjusted model are: rs9391858 (*C6orf10*, 1.7 × 10^-33^), rs660895 (*HLA-DRB1*, 7.7 × 10^-19^), rs2395175 (*HLA-DRA*, 5.1 × 10^-16^), rs2524123 (*HLA-C*, 9.3 × 10^-8^), and rs3130237 (*COL11A2*, 1.62 × 10^-8^). From the same model, the variable indicating the number of high-risk *HLA-DRB1 *alleles and the variable indicating the number of other risk alleles had *p*-values of 0.017 and 0.79, respectively. After applying this adjusted model on all other chromosome 6 SNPs, the most significant SNP was rs11964472 (*p*-value 1.8 × 10^-6^), located near the *PAQR8 *and *MCM3 *genes on 6p12.

Pair-wise interaction tests among these four SNPs and the number of *HLA-DRB1 *risk alleles indicate that no significant interaction exists among the four SNPs in the adjusted model or the number of *HLA-DRB1 *risk alleles.

### Enrichment of interaction effects among SNPs with significant main effects

We sought to find significant SNP × SNP interactions among the SNPs with significant main effects after adjustment for the number of high-risk *HLA-DRB1 *alleles, the number of other risk alleles, and the five SNPs near the *HLA-DRB1 *locus. Of the genome-wide SNPs passing quality control filters, 178 had main effect *p*-values < 10^-4^.

This set of SNPs was significantly enriched with interaction *p*-values < 10^-4 ^based on permutation tests. We performed 15,383 unadjusted pair-wise interaction tests with these SNPs. Using a Bonferroni correction, the *p*-value cut-off for significance would be 0.05/15,383 = 3.25 × 10^-6^. Of the 15,383 unadjusted pair-wise tests, two tests had *p*-values < 3.17 × 10^-6 ^with a permutation *p*-value of 0.003, indicating significant enrichment of interaction effects in this set of SNPs. In the adjusted models, which adjust for the strong signal in *HLA-DRB1*, the most significant interaction effect occurred between rs1333914 in *DBC1 *on chromosome 9 and *C6orf10 *on chromosome 6, with an interaction *p*-value of 5.7 × 10^-5^. The top ten results of the adjusted pair-wise interaction analyses are presented in Table 1.

**Table 1:** Top ten pair-wise interaction results among SNPs with main-effects *p*-values < 1 × 10^-4 ^from logistic regression models

SNP 1	Chr	Position (1,000 bp)	Closest gene	SNP 2	Chr	Position (1,000 bp)	Closest gene	Interaction *p*-value
rs1333914	9	121623	*DBC1*	rs9391858^a^	6	32449	*C6orf10*	5.7 × 10^-5^
rs9368950	6	36736	*CDKN1A*	rs9408926	9	122955	*CEP110*	5.7 × 10^-5^
rs3873444	6	32791	*HLA-DQA2*	rs17172185	7	43253	*HECW1*	1.1 × 10^-4^
rs9368950	6	36736	*CDKN1A*	rs9408928	9	122992	*RAB14*	1.9 × 10^-4^
rs7679231	4	137102	*PCDH18*	rs8017455	14	80645	*TSHR*	2.4 × 10^-4^
rs532098	6	32686	*HLA-DRB1*	rs660895^a^	6	32685	*HLA-DRB1*	2.6 × 10^-4^
rs4674520	2	201422	*CLK1*	rs2524123^a^	6	31373	*HLA-C*	3.3 × 10^-4^
rs10948693	6	52262	*MCM3*	rs7330752	13	29755	*KATNAL1*	3.8 × 10^-4^
rs4674520	2	201427	*CLK1*	rs10948693	6	52262	*MCM3*	4.0 × 10^-4^

^a^SNPs from the adjusted model

### Enrichment of main and interaction effects among SNPs in gene collections

Using sets of SNPs in biologically derived gene collections, we used permutations to establish the significance of the proportion of main effects and interaction effects below certain *p*-value thresholds. The results of this analysis are presented in Table 2. Of interest are two gene collections that contain the *TRAF1 *gene. SNPs near *TRAF1 *account for the most significant signals in the GWAS (rs2900180, *p *= 2.1 × 10^-9^) after adjusting for the *HLA-DRB1 *locus. The "small cell lung cancer" KEGG pathway, which contains the *TRAF1 *gene, is significantly enriched with significant main effect *p*-values, but not with significant interaction effects. The enrichment of main effects *p*-values is driven by the significant SNPs in the *TRAF1 *gene. On the other hand, the "protein complex assembly" set of SNPs from GO is significantly enriched with both significant main effects and interaction effects. Two genes in this set, *TRAF1 *and *TAP2*, have significant main effects after adjusting for the *HLA-DRB1 *locus, which is very close to the *HLA-DRB1 *locus. Among the SNPs within 60 kb of the *TAP2 *gene, unadjusted interaction *p*-values were as low as 1 × 10^-20^. Such high significance is most likely due to the unadjusted nature of the tests for interaction using the--fast-epistasis command in PLINK: SNPs within *TAP2 *capture the strong signal present in the region.

**Table 2:** Number of tests with *p*-values < 10^-6^, 10^-7^, 10^-8^

						No. *p*-Values below threshold^a ^(permutation *p*-value)		
						
SNP set	Source	RA gene included in set	No. genes in set	Test^b^	*N *tests	<10^-6^	<10^-7^	<10^-8^
Small cell lung cancer	KEGG	TRAF1	87	Main effects	3,429	6 (<0.001)	6 (<0.001)	3 (<0.001)
				Interaction	5,800,955	8 (0.081)	0 (1)	0 (1)
JAK-STAT signaling pathway	KEGG	STAT4	153	Main effects	3,253	0 (1)	0 (1)	0 (1)
				Interaction	5,169,188	7 (0.137)	3 (0.029)	0 (1)
Protein complex assembly	GO	TRAF1	214	Main effects	3,883	6 (<0.001)	6 (<0.001)	3 (<0.001)
				Interaction	7,277,905	292 (0.642)	193 (0.152)	151 (0.012)
Protein amino acid dephosphorylation	GO	PTPN22	73	Main effects	2,277	0 (1)	0 (1)	0 (1)
				Interaction	2,496,978	1 (0.735)	0 (1)	0 (1)
JAK-STAT cascade	GO	STAT4	41	Main effects	761	0 (1)	0 (1)	0 (1)
				Interaction	289,031	0 (1)	0 (1)	0 (1)
Activation of MAPK activity	GO	C5	47	Main effects	785	3 (<0.001)	3 (<0.001)	1 (<0.001)
				Interaction	307,355	0 (1)	0 (1)	0 (1)
Chemotaxis	GO	C5	112	Main effects	1,470	3 (<0.001)	3 (<0.001)	1 (<0.001)
				Interaction	1,065,328	1 (0.467)	0 (1)	0 (1)
Inflammatory response	GO	C5	180	Main effects	2,535	3 (<0.001)	3 (<0.001)	1 (<0.001)
				Interaction	3,163,945	8 (0.025)	0 (1)	0 (1)

^a^Empirical significance was determined by comparing the number of *p*-values below each threshold to a null distribution of the number obtained by permuting the phenotypes and performing main effects and interaction tests with the permuted data set.

^b^Interaction tests are unadjusted.

We subsequently studied the pair-wise interaction between SNPs in the "protein complex assembly" gene collection using logistic regression models that adjusted for the *HLA-DRB1 *locus. Because there are a large number of genes and SNPs in this collection (214 and 3,883 respectively), we filtered the number of SNPs by main effects *p*-values = 0.20 and *r*^2 ^> 0.5, for a total of 572 SNPs. The top ten results observed among these tests are presented in Table 3.

**Table 3:** The top ten pair-wise interaction results among SNPs near genes in the "protein complex assembly" gene collection from a logistic regression model

SNP 1	Chr	Closet gene	SNP 2	Chr	Closet gene	Interaction *p*-value
rs12969200	18	*DSG1*	rs12458537	18	*MALT1*	1.05 × 10^-7^
rs215590	16	*MYH11*	rs9391858^b^	6	*C6orf10*	1.86 × 10^-6^
rs13035837	2	*DGKD*	rs2672221	11	*NUP98*	3.27 × 10^-6^
rs335524	1	*CENPF*	rs215590	16	*MYH11*	1.31 × 10^-5^
rs10834677	11	*ART5*	rs17286488	16	*MYH11*	2.21 × 10^-5^
rs1553920	1	*PIK3C2B*	rs6888329	5	*ADRB2*	5.38 × 10^-5^
rs9311951	3	*MAGI1*	rs2191498	7	*CAV2*	5.64 × 10^-5^
rs774265	7	*SKAP2*	rs1412243	9	*APBA1*	8.64 × 10^-5^
rs10739575	9	*PSMD5*	rs1805061	14	*SLC7A7*	9.88 × 10^-5^

^a^SNPs were filtered by main effects *p*-values = 0.20 and binned by *r*^2 ^> 0.5, for a total of 572 SNPs

^b^SNPs from the adjustment model

## Discussion

We found that the set of SNPs with significant main effects was enriched with significant interaction effects, using an interaction *p*-value cut-off of 3.17 × 10^-6^. In using biological information from the KEGG and GO databases, significant enrichment of low-interaction *p*-values was observed when genes such as *TRAF1 *and *TAP2 *were included in the gene sets. These genes were close to the most significant regions in the GWAS. None of the other gene sets were found to be enriched with significant main effects or pair-wise interactions.

Despite these promising findings, both methods of selecting SNPs for tests of interactions have disadvantages. When selecting SNPs with significant main effects, those interactions among SNPs without main effects will be missed. The justification for the approach is the gain in power by testing fewer SNPs. The power to detect interaction in the absence of main effects is very low when tests of interaction are performed in the entire genome due to the number of tests being performed. In the selection of SNPs using "expert knowledge" from biological databases, the quality of the gene sets is only as good as the quality of the database. The curation of these databases is a developing science and there are many reasons to believe that the quality of these resources and the data they provide will continue to improve.

## List of abbreviations used

GAW16: Genetic Analysis Workshop 16; GWAS: Genome-wide association studies; RA: Rheumatoid arthritis; GO: Gene ontology; KEGG: Kyoto Encyclopedia of Genes and Genomes; SNP: Single-nucleotide polymorphism

## Competing interests

The authors declare that they have no competing interests.

## Authors' contributions

AKM, JSN, JD, and LAC conceived of this study. The data were cleaned by AKM and AEH. The analysis was carried out by AKM and JSN. Gene annotations and gene databases were provided by ADJ and C-TL. The manuscript was written by AKM. All authors contributed toward reviewing and revising the manuscript.
